# Biologically inspired warning patterns deter a passerine, *Parus major*, from digital turbine blades

**DOI:** 10.1093/beheco/arag039

**Published:** 2026-04-20

**Authors:** George R A Hancock, Heta Lehtonen, Theo Brown, Amine Ejjite, Ossi Nokelainen, Johanna Mappes, Sandra Winters

**Affiliations:** Organismal and Evolutionary Biology Research Programme, Faculty of Biological and Environmental Sciences, University of Helsinki, Helsinki 00014, Finland; Centre for Ecology & Conservation, University of Exeter, Penryn TR10 9FE, United Kingdom; Organismal and Evolutionary Biology Research Programme, Faculty of Biological and Environmental Sciences, University of Helsinki, Helsinki 00014, Finland; Organismal and Evolutionary Biology Research Programme, Faculty of Biological and Environmental Sciences, University of Helsinki, Helsinki 00014, Finland; Lammi Biological Station, Pääjärventie 320, Lammi 16900, Finland; Organismal and Evolutionary Biology Research Programme, Faculty of Biological and Environmental Sciences, University of Helsinki, Helsinki 00014, Finland; Department of Biological and Environmental Science, University of Jyväskylä, Jyväskylä 40014, Finland; Open Science Centre, University of Jyväskylä, Jyväskylä 40100, Finland; Organismal and Evolutionary Biology Research Programme, Faculty of Biological and Environmental Sciences, University of Helsinki, Helsinki 00014, Finland; Organismal and Evolutionary Biology Research Programme, Faculty of Biological and Environmental Sciences, University of Helsinki, Helsinki 00014, Finland

**Keywords:** biomimicry, bird conservation, collision mitigation, vision, warning signals

## Abstract

Wind power has been at the forefront of renewable energy investment, but bird fatalities from turbine collisions remain a key ecological and social concern. Increasingly, how principles from sensory ecology might reduce collisions by enhancing the detectability or aversiveness of turbine blades have been investigated. In nature, aposematic species use high-contrast colors and striped patterns to warn predators of their unprofitability. These signal elements are effective due to their conspicuousness across variable natural scenes, memorability, generalisability from mimicry, and exploitation of innate color aversions. This begs the question: might employing biologically inspired turbine warning colors help birds to avoid turbine blades? Here, we used a screen-based “game” experimental setup to test the behavioral responses of wild-caught great tits (*Parus major*) to 3 existing wind turbine patterns (white, red-striped, and single black blade) as well as a novel biologically inspired aposematic pattern. Tits were less likely to approach and, when they did approach, took significantly longer to approach patterned than uniform white blades. This effect was strongest for our bio-inspired pattern. Our work supports the need for further investigation into the use of warning patterns to reduce bird collisions with wind turbines.

## Teaser

By mimicking the warning colors of animals that ward off birds, we can produce wind turbine patterns to which birds are averse, reducing the likelihood of collisions. This is a valuable intervention as, although wind turbines are an invaluable source of renewable energy, they pose a hazard to endangered birds.

Impact Statement Bio-inspired warning patterns make birds more aversive of wind turbines. Applying these patterns should be considered to protect birds.

## Introduction

Rising international concern over the ecological and economic impacts of climate change and the resulting race to limit global warming to <2 °C above preindustrial levels has led to increasing investment and research into low-carbon energy sources to help transition away from fossil fuels (Mitigation 2011; Agreement 2015). Of the energy alternatives, wind power has been identified as one of the most promising energy sources due to its wide geographic applicability and relative efficiency (Lu and McElroy 2023). However, the construction of wind power plants can displace species (Zwart et al. 2016; Tolvanen et al. 2023) and lead to fatalities either from collisions between flying animals and turbines (Johnson et al. 2003; Marques et al. 2014; Schuster et al. 2015) or by forming sensory traps from attraction to the turbine's color, as is the case for invertebrates (Long et al. 2011; Voigt 2021). Bird fatalities from wind turbines occur from collisions with the moving turbine rotor blades or the static tower (Loss et al. 2015; Stokke et al. 2020). Blade collision risk is higher for birds that soar at the same altitude as the blades, such as eagles and vultures (Rushworth and Krüger 2014; Perrow 2017; Watson et al. 2018), and birds that migrate through wind farms in large numbers and/or at night (Richardson 1998). Tower collisions, on the other hand, have almost exclusively been observed from low-flying ground-nesting birds, such as willow ptarmigan (*Lagopus lagopus*), black grouse (*Lyrurus tetrix*), and capercaillie (*Tetrao urogallus*) (Zeiler and Gruenschachner-Berger 2009; Gonzalez et al. 2016; Coppes et al. 2020; Stokke et al. 2020). Despite efforts to strategically place wind turbines within lower-risk sites, accelerating expansion of wind turbine deployment will only increase the conflict between energy production and the environment (Balotari-Chiebáo and Byholm 2024), inciting the need to develop effective mitigation strategies for collisions to preserve protected species.

The question of why birds collide with objects that are highly conspicuous to the human eye challenges our understanding of how birds identify potential hazards, how their visual systems differ from those of humans, and, importantly, how to resolve this issue. Several factors have been cited as likely causes for bird collisions with wind turbine blades, including failure to detect the wind turbine blades due to low-light conditions (Hüppop et al. 2006), low visual acuity (Martin and Banks 2023), motion smear from the high blade tip speeds, and limited field of view (Hodos et al. 2001; May et al. 2020). Additionally, birds could fail to recognize turbines as a threat (Simmons and Martins 2024) or may be unable to maneuver safely away from them when within the blade-swept zone (De Lucas et al. 2008; Marques et al. 2014). However, the infrequency of behavioral observations of birds at the time of collision with turbines has made it difficult to identify the cause. Proposals to reduce the risk of bird collisions with wind turbines have increasingly relied upon knowledge of the sensory and behavioral ecology of the species at risk. These include using auditory deterrents for bats or birds (Smith et al. 2011; Arnett et al. 2013) and visual deterrents such as modifications to the wind turbines themselves with reflectors, flashing lights, or painted patterns to increase their conspicuousness (Kerlinger et al. 2010; May et al. 2020; Stokke et al. 2020).

Painting turbines has been highlighted as one of the best possible options for a passive visual deterrent due to its lower dependence on lighting than reflectors, lower likelihood of attracting nocturnal birds compared with flashing lights (Kerlinger et al. 2010), and lower running cost compared with alternative ancillary detection technology from radar or camera-based systems (Marques et al. 2014; Singh et al. 2015). Within the natural world, warning signals—which can be visual, acoustic or olfactory—are commonly used by animals to signal themselves as being unprofitable to attack (Mappes et al. 2005; Caro and Ruxton 2019). This phenomenon, known as aposematism, is thought to explain the prevalence of conspicuous colorations within numerous unpalatable or “dangerous” taxa, such as poisonous frogs, caterpillars, and fish, venomous snakes, and spined sea urchins, to name a few examples. As signals are often more effective when they are more easily recognized and remembered by the predator (Endler and Rojas 2009; Ruxton et al. 2019), aposematic species have frequently evolved colors and patterns that are salient against the variable conditions of natural backgrounds (Sillén-Tullberg 1985; Aronsson and Gamberale-Stille 2009; Stevens and Ruxton 2012) and are similar to those of other local unpalatable species (Müllerian mimicry) (Beatty et al. 2004; Sherratt 2008). Most terrestrial aposematic colors are likely to be directed at birds given their prevalence as predators (Kassarov 2003) and the commonly converged evolution of “bright” longwave (red, orange, and yellow in human vision) colors, which are less easily distinguished from many natural backgrounds to dichromatic mammalian observers (Lawrence and Noonan 2018). Behavioral experiments with birds, namely Passerines and Galliformes, have shown that they can quickly and socially learn to avoid warning colors (Roper and Wistow 1986; Aronsson and Gamberale-Stille 2008; Landová et al. 2017; Lawrence and Noonan 2018; Thorogood et al. 2018) and possess unlearned aversion towards longwave colorations and highly achromatically contrasting patterning (eg black stripes and spots), common in aposematic species (Schuler and Hesse 1985; Lindström et al. 1999; Pegram and Rutowski 2014; Halpin et al. 2020; Protti-Sánchez et al. 2023). Species particularly vulnerable to collisions with turbines, such as raptors, have also been shown to avoid attacking aposematic patterns of sympatric prey species, such as those of venomous and nonvenomous Batesian mimetic viperid and elapid snakes (Brodie 1993; Valkonen et al. 2011). While smaller birds, such as Passerines, also collide with wind turbines, but are often under-sampled (Nilsson et al. 2023). Consequently, Passerines—while not the primary collision-prone group—provide a tractable experimental system for identifying color patterns with potential relevance for increasing turbine visibility or providing a warning to raptors and birds in general.

**Figure 1:**
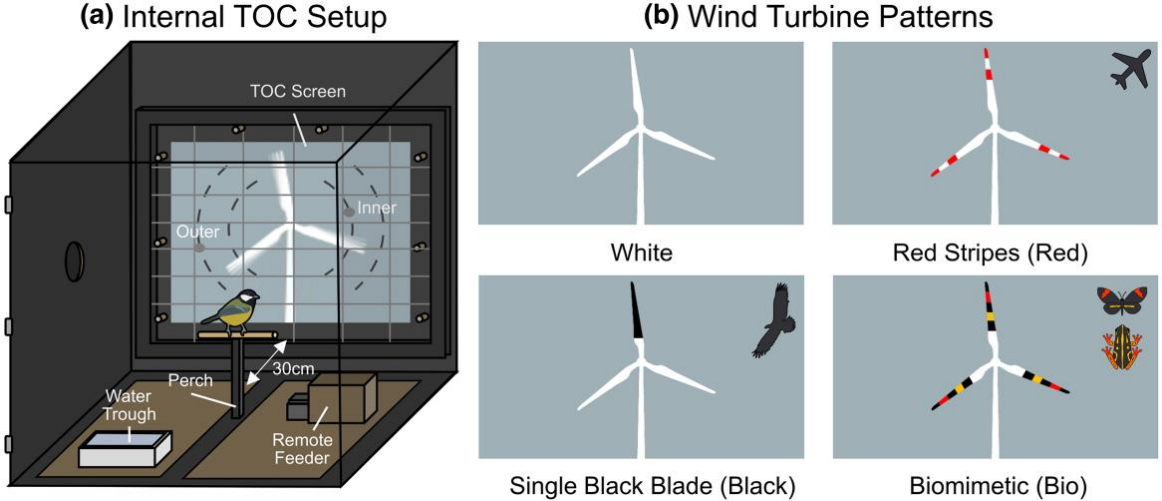
a) Illustration of internal touch operant chamber (TOC) setup. Wind turbine stimuli during the final experiment were presented on the screen with 2 target dots, 1 inside the rotor-swept area of the turbine blades (inner) and the other outside (outer). The dashed lines show the possible locations where the inner and outer dots could occur. b) Wind turbine stimuli are shown to scale with their backgrounds and the inspiration for their function shown on the right for the 3 patterned wind turbines (red = aviation warning, black = raptor deterrent, and bio = inspired by warning signals).

Despite the prevalence of aposematic coloration, using warning signals on wind turbines has seen limited experimental investigation and no study has used patterns directly inspired by aposematic animals. By using biomimicry (ie, the act of copying nature to solve problems) we might be able to design wind turbine patterns that reduce the likelihood of collisions with the blades. As most birds are tetrachromats and are able to detect varying degrees of UV light, increasing the UV reflectance of wind turbines from their typical 10% UV reflectance (UV dark) to 70% (white) has been field trialed as a cryptic warning (invisible to humans), with inconclusive results (Erickson et al. 2003). Painting both the base of the tower and a single blade of the turbine black has been shown to reduce collision risk for grouse and raptors, respectively (May et al. 2020; Stokke et al. 2020). In both cases, black was thought to be more conspicuous against the background than white, and in the latter case, painting a single blade helps to reduce motion smear based on electroretinogram responses to blades within a laboratory setting (Hodos 2003). Meanwhile, contrasting red stripes are already applied to wind turbines in some countries, such as Germany (Fig. 1), to signify their proximity to airstrips. Given the prevalence of black and red in aposematic signals (Robinson et al. 2023), these patterns may also provide a warning function, increasing the aversion of birds to the turbines, but this remains untested.

Here, we aimed to test how birds approach digital model turbines with different blade pattern designs. Specifically, we provide the first behavioral comparison of avian approach time and likelihood to both approach and to choose a target within the rotor swept zone of these digital model turbines with existing blade patterns (white, red-striped, and single black blade) and a novel biomimetic pattern inspired by aposematic animals, which includes, as perceived by humans, red, yellow, and black stripes. To do this, we used touch screen-based behavioral assays where a single model species, the great tit (*Parus major*), was tasked with pecking 2-dimensional gray dot-shaped targets inside and outside the radius of the digital wind turbine blades rotating at a speed within the typical average range of turbines, 20 rpm, in exchange for food (May et al. 2012; Blary et al. 2023). We predicted that the biomimetic pattern should decrease the likelihood and increase the latency of birds pecking the targets, particularly those inside the radius of the blades, where the real-world collision risk would be higher.

## Methods

### Experimental setup

For our experiments, we used wild Eurasian great tits (*Parus major*, *n* = 22) captured and housed at Lammi Biological Research Station (61.0543N, 25.04086E) as a model avian observer. Great tits were chosen, due to their established use for avian perceptual and cognitive research, their trainability, adaptability to aviary conditions, and the ability to use wild-caught rather than captive birds. The nearest wind turbine site was over 80 km away, so the birds were unlikely to have regularly encountered wind turbines before and thus, having any prelearned response on our experimental set-up (Greenwood et al. 1979). Birds were captured using a trapping system, housed individually throughout our experiments, and provided with food and water ad libitum, except during training and testing, where food was deprived prior to and returned after, to ensure motivation.

During initial training, birds were housed in temporary home aviaries (46 × 68 × 58 cm) before being transferred to 1 of 3 larger aviaries (90 × 90 × 79 cm), which also acted as our touchscreen operant chambers (TOCs) (Fig. 1; Seitz et al. 2021; Winters et al. 2026). Both the temporary home aviaries and the larger TOC aviaries were illuminated with warm nonUV emitting LED lighting, which operated on a 12-h light and a 12-h dark cycle between 7 AM and 7 PM with all experiments taking place under diurnal conditions between 9 AM and 4 PM. All aviaries were custom-built from plywood and contained a perch for the birds to sit and sleep on. Use of birds for our behavioral experiments was conducted with approval from the Central Finland Center for Economic Development, Transport and Environment (permit no. 6870/2022). All birds were released into the area where they were caught once they had completed the experiment and had no indication of ill effects from handling or training. Birds were housed for no longer than 2 wks, with training and experimentation typically taking between 5 and 7 d total.

The TOCs used high-performance GPU-enabled PCs (Lenovo Legion T5 Ryzen 7 with 16GB RTX 3070) and gaming monitors (Asus ROG Swift PG329Q 32″ at 165 Hz, 2560 × 1,440 resolution) to present moving stimuli with a frame rate higher than the critical flicker fusion frequency (CFF) of the most closely related species for which the CFF is known, the blue tit (*Cyanistes caeruleus*) which sees at up to a maximum of 131 Hz (Boström et al. 2016). Each screen was calibrated using a Calibrite ColorChecker Studio (XRITE, Grand Rapids, MI, USA). To allow the birds’ beaks to interact with the screens we used an infrared touch frame (G6 Integration Kit Touch Frame 6TP 32″, 250 fps maximum). As the birds were unable to navigate the screen by flight alone (eg, as some hovering birds can do), a steel grid mesh was positioned in front of the IR frame to allow the bird to access the entire screen area. A 3 mm thick acrylic sheet cut to the same dimensions as the monitor was placed between the infrared frame and the screen to protect it from damage. All digital stimuli were rendered using the Psychtoolbox-3 for MATLAB (Kleiner et al. 2007). All MATLAB code and images used to construct the digital stimuli are available within our Supplementary material. A full breakdown of our TOC apparatus can be found in (Winters et al. 2026). Bird training and experiments were carried out between January and April 2024.

### Turbine design

Each turbine consisted of a live 2D render against a blue–gray background (159, 176, 183 RGB; chosen to approximate the color of an overcast Finnish sky) with a 20 cm tower and 16 cm blades (Fig. 1). The blade shapes were constructed by tracing real-world images of wind turbines, and for each turbine, the white regions were (255, 255, 255 RGB). We used 4 turbine designs: all white (255, 255, 255 RGB; “white”), 2 red (255, 0, 0 RGB) stripes on the outer portions of all blades (“red”), a single black (0, 0, 0 RGB) blade (“black”), and a biomimetic design (“bio”). For the biomimetic blade pattern, we used a, as perceived by humans, “red” (255, 0, 0 RGB), “yellow” (255, 192, 0 RGB), and “black” striped pattern along each blade. For the purposes of avian vision, the red color would have equated to a more longwave dominant color than the yellow. For this pattern, we included common signal components of aposematic visual signals: long-wavelength colors, high luminance contrast, and stripes (Fig. 1) (Brodie 1993; Exnerová et al. 2003; Valkonen et al. 2011; Stevens and Ruxton 2012). For our biomimetic pattern, we picked a spatial scale of stripes that was both equal to the red stripe pattern of the “red” turbine (1.91 cm) and which would be visible when static from the maximum viewing distance of the TOC (100 cm) when modeled with the acuity of similarly sized members of the family Paridae (5 cp/d) (Moore et al. 2013; Caves et al. 2024). As neither the screens nor lights used for the aviaries emitted UV light, the white blades would have been similarly UV dark to actual wind turbines, although the background would have lacked the high proportion of UV light within a natural sky. Due to the use of an RGB screen, the spectra used were not and could not be matched to any 1 species, due to the limited longwave length gamut and the absence of UV, although the colors of aposematic species are also frequently UV dark, especially those which humans perceive as red (Lyytinen et al. 2001; Barnett et al. 2018; Crowell et al. 2024).

### Initial training

Before being exposed to wind turbines, the birds were trained to peck 1.75 cm dark gray dot stimuli in exchange for a food reward (Silvasti et al. 2021). Training birds to reliably peck a target stimulus allowed us to then identify how turbine appearance and relative location influenced their willingness to do so. Gray dots were chosen as a generic neutral stimulus with a luminance and chromatic value distinct from the values used for the turbines. First, birds were trained to associate a gray printed paper dot with food by placing a sunflower seed in a slit inside the dot to be retrieved. Dots were presented in 2× palettes of 6 (12 dots) following methods previously used to train tits to attack digital stimuli (Silvasti et al. 2021). After the birds had successfully fed from all of the dots, the seeds were then glued underneath the dots for another 2× palettes of 6 such that the birds had to peck and rip through the dot to access their food. Birds were then trained to feed from an open food reward box containing half-cut sunflower seeds before being moved to one of the TOC aviaries. Next, they were then trained to associate pecking at a printed dot with the food reward box opening remotely and retrieving food from it. These physical dots were then gradually moved further from the feeder until the bird was able to peck them on the screen against the blue–gray background. As some birds struggled to peck at dots on the screen a Perspex cover was placed over the printed dot for some of the trials to ensure they would still try to peck the dot even if they could not physically touch it. Lastly, birds were trained to peck at a digital dot (114, 114, 114 RGB) against the blue–gray background (159, 176, 183 RGB) used for the turbines on the touchscreen instead of a physical dot. Birds were also trained to associate landing on the central perch and facing the screen with activation of the trials. Once the birds had successfully pecked dots in all 4 quarters of the screen, 5 times each and in <2 min per dot, they were allowed to move on to the experiments. 10 of the 32 birds captured failed to finish the initial training within 5 d (69% success rate) and were released and ringed to prevent recapture.

### Turbine training

Pilot testing showed that naive birds were unwilling to approach a novel turbine stimulus with blades spinning at full speed, necessitating a training stage to facilitate acceptance of this novel stimulus. To prevent potential confounds that could arise from training on alternative turbine appearances, each bird was randomly assigned 1 of the 4 wind turbine patterns for training (white *N* = 5; red = 5; black *N* = 6; bio *N* = 6); in later experimental trials, this training turbine pattern was therefore “familiar”, and the other 3 patterns were “novel”. For each turbine training trial, birds were tasked with pecking a single gray dot in the presence of turbines rotating at various speeds (0, 2.5, 5, 10 rpm), starting with a stationary turbine (0 rpm), then proceeding through additional speed increases in order (See Supplementary Video TurbineSpeeds). Birds had to peck the dot within 2 min of it appearing. The dot could appear along an arc to the right or left of center of the rotor, either inside (radius = 14 cm from center, inner) or outside (radius = 20 cm from center, outer) of the rotor-swept area of the turbine blades. Inner targets were rendered behind the blades (ie, the blades covered the dot as they rotated). Each bird had to successfully peck 5 inner and 5 outer dots for each speed before advancing. Once the dot was clicked, the screen turned gray (127, 127, 127 RGB) and a food reward was given. Dots were randomly ordered such that an inner or outer dot could not occur more than twice in a row. The starting orientation of the blades was randomized for each trial and the blades always spun clockwise. The number of trials (5 per dot type) was determined from pilot observations for the minimum needed to obtain stable approach behavior across multiple target positions, while keeping total training time within 2 to 3 d to minimize captivity duration. For each trial, we recorded the bird ID, time, date, order (trial number for that bird), time taken for the bird to peck the dot, whether the dot was in/out, left/right, and whether the bird timed out. Trials were monitored by the experimenter using a viewing window and recorded using a camera mounted inside the TOC aviary (GoPro Hero11 Black, San Mateo, California, United States). In some trials, an issue with the touch-frame encoding for the Psychtoolbox (later resolved, see Supplementary material) prevented a peck on the dot from registering; in these cases, a key press override was instead used by the experimenter to trigger the end of the trial. The time between the onset of the trial and the peck on the dot was recorded by MATLAB and with a stopwatch as backup. Trials were typically shown in blocks of 4 to 6 trials back-to-back depending on how long the birds maintained motivation, with 20 to 30 min breaks in between each block. Birds that completed trials but did not take any food were considered to no longer be food-motivated and were given a break. Birds that completed the criterion at 10 rpm proceeded to the blade color comparison experiment.

### Blade color comparison experiment

In the blade color comparison experiment, we assessed the responses of each bird to each of the 4 turbine patterns. Before the experiment commenced, each bird was presented with a series of control trials using the same wind turbine pattern it was trained with (“familiar” pattern). Unlike the training experiment, for the control trials, both an inner and an outer dot were shown simultaneously, and the speed of the blades was doubled once more, this time from 10 to 20 rpm (see Supplementary Video TurbineSpeeds). The birds could now peck either the inner or the outer dot, resulting in the same gray screen and food reward once one of the dots was pecked. We recorded the dot they selected (inner or outer), then continued the trial after the bird had eaten and resumed its position on the perch, with the same stimulus being shown again but with only the un-pecked dot remaining. Each comparison trial with a given turbine appearance therefore consisted of 2 (potential) pecks at dots, with a brief break in between. Birds had to complete a minimum of 4 control trials (8 dots), with 3 successful trials in a row before moving on to the final comparison experiment. A trial was considered successful if at least 1 of the 2 dots was pecked. Once the control trials were completed, the birds were exposed to all 4 blade patterns, including the familiar training pattern and 3 novel ones. Trials were conducted in 5 blocks of 4, with each block consisting of the 4 turbine patterns, randomly ordered using pregenerated Latin squares. After completing the experiment birds were released and color-ringed to prevent recapture. On average initial training, turbine training and the comparative experiment room a total of 5 d (see Supplementary Training Log for full list of training dates), with all phases of the comparative experiment being completed within the span of 1 d.

### Statistical analyses

For our analyses, we compared the effect of the wind turbine patterns and target positions (outer = low risk & inner = high-risk) on bird behavior with R version 4.3.2 (R Core Team 2023) using linear mixed models (LMMs) and binomial generalized linear mixed models (GLMMs) with the “lme4” package (Bates et al. 2015). For the training experiment, we tested whether the turbine pattern and target position as well as the (factorial) speed of the blades and the trial number influenced the proportion of timeouts (when birds did not peck a dot within 2 min; binomial model) and the capture time for successful target pecks (linear model). This approach first assesses what affects birds’ willingness to approach the turbine, then for birds that do approach, what affects their peck latency. For the comparison experiment, in addition to measuring the effects of pattern and target position on capture time (linear model) and the proportion of timeouts (binomial model) we also tested the effect of novelty whether the blade pattern shown was 1 it was trained with (familiar) or a novel pattern (novel), trial number (1 to 20), and whether the trial was the first or second dot pecked. We also tested the effect of these predictor variables on the proportion of outer dots chosen first (binomial model) using a subset of the data that only included the first part of each trial (ie, the first out of 2 dot presentations). To compare differences between factor levels (eg, wind turbine color or speed), we used emmeans Tukey post hoc tests (https://CRAN.R-project.org/package=emmeans).

For each test, we constructed a base model containing all fixed effects and then used likelihood ratio tests to remove nonsignificant terms from the model in a stepwise fashion. Retained variables were then tested for significant interactions by comparing the fit of models with different levels of interactions to a model without interactions. For each model, the ID of the bird was included as a random effect. For the comparative experiment, trial ID (trial number + bird ID) was used as a random effect, as each trial contained 2 dot pecks. For the training experiment, day (trial date–earliest date) was included as a random effect. Day was not included for the comparative experiment as all trials were completed on the same day. For comparisons of capture time, the log time was used as it was closer to a normal distribution than the raw time. For all models, numeric variables were rescaled to a mean of zero and a standard deviation of 1. Residual plots were used to verify that all models met assumptions.

## Results

### Turbine training exercises

**Figure 2:**
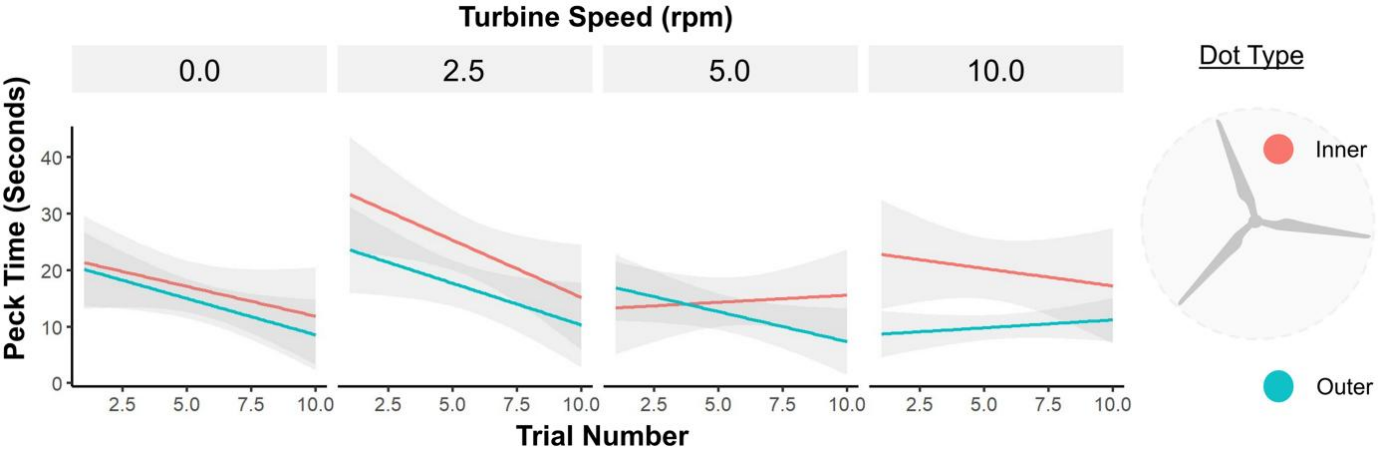
Effect of trial number, turbine speed and dot type, on the time taken for birds to peck the dot in seconds. Lines show the fitted linear model for the inner “higher risk” dot (red) and the outer “lower risk” dot (blue). The gray bars show the standard error.

**Figure 3:**
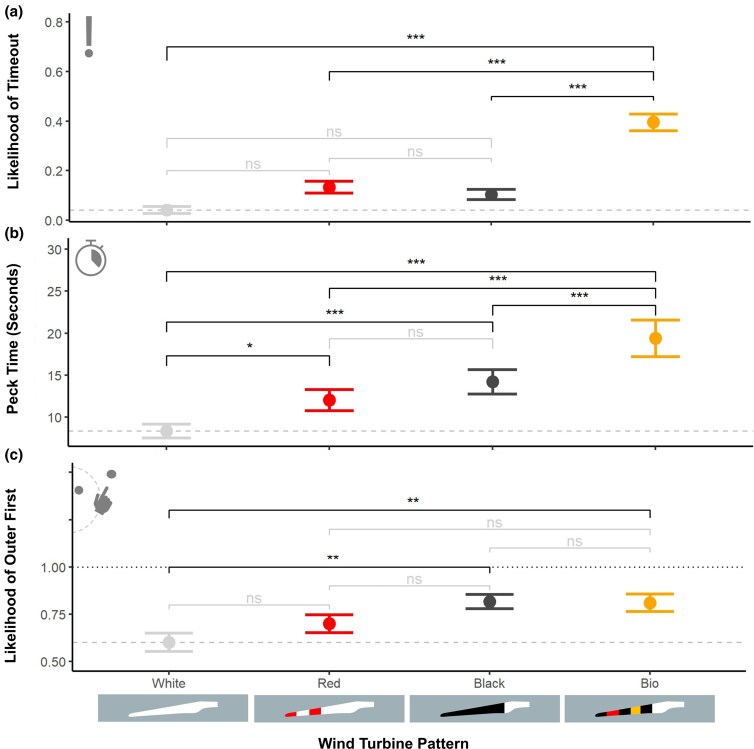
a) The effect of turbine blade pattern on the likelihood of a bird timing out during a trial, greater values indicate a greater likelihood of a timeout. b) The effect of turbine blade pattern and pattern novelty (familiar = trained with, novel = not trained with) on the log time for the target dot to be pecked. c) The effect of turbine blade pattern on the binomial for dot choice, greater values indicate a greater likelihood of choosing the outer dot first (lower risk). For all plots, points show the mean and the error bars show the SE. Brackets indicate the significance of Tukey post hoc comparisons between blade patterns, where ns = not significant, *P < 0.05, **P < 0.01, and ***P < 0.001. The dashed gray lines indicate the mean for the white un-patterned turbines.

For the training results, only the first 10 trials for each speed were used in our analyses as some bird’s required additional trials to meet the criteria of 5 successes for inner dots and 5 successes for outer dots. For the binomial model assessing timeouts, only speed and trial number were retained as effects, meanwhile for log capture time, speed, dot type and trial number were retained. Blade pattern had no significant effect on timeouts or capture time during training. However, birds on average did take longer to respond to the appearance of dots for patterned blades (red, black and bio) compared with white when training commenced (speed = 0, see Supplementary material). Later trials were significantly less likely to timeout (*β* = −0.173, *z* = −3.500, *P* < 0.001) and had shorter capture times (*β* = −0.0314, *t*_752.9_ = −2.760, *P* = 0.001) as the birds became more habituated to the blades. Birds took significantly longer to peck outer dots compared with inner dots (*β* = −0.210, *t*_752.54_ = −3.242, *P* = 0.001). The number of timeouts did not increase across speeds as was expected. Post Hoc comparisons between speeds showed that birds were significantly more likely to timeout at 2.5 rpm compared with 0 rpm (Table 1) and took significantly longer to capture dots for 2.5 rpm compared with all other speeds (Fig. 3a, Table 1). This was likely in response to the novelty of motion (2.5 is the first speed jump after 0) as capture time declined with repeated exposure to moving blades (Fig. 2).

**Table 1:** Results for emmeans Tukey posthoc comparisons of wind turbine speed's effect on the likelihood of a timeout and capture time during the turbine training exercise.

Speed A	Speed B	Timeout				Capture time			
		Estimate	SE	*Z* value	*P* value	Estimate	SE	*t* value	*P* value
0.0 rpm	2.5 rpm	−1.484	0.415	−3.574	0.002	−0.382	0.092	−4.019	<0.001
0.0 rpm	5.0 rpm	−0.831	0.483	−1.719	0.313	0.0877	0.091	0.730	0.8844
0.0 rpm	10.0 rpm	−0.613	0.529	−1.159	0.653	0.0104	0.090	0.077	0.9998
2.5 rpm	5.0 rpm	0.653	0.381	1.716	0.315	0.470	0.090	4.312	<0.001
2.5 rpm	10.0 rpm	0.871	0.423	2.060	0.166	0.393	0.065	3.250	0.0139
5.0 rpm	10.0 rpm	0.218	0.415	0.524	0.953	−0.077	0.011	−0.820	0.845

Negative estimates indicate that speed A has a lower likelihood of a time out/capture time than B, while positive values indicate that A is higher than B.

### Blade color comparison experiment

Of the 440 trials conducted, 14 were excluded due to the bird not engaging in the trial (eg, bathing, preening, or other signs of inattention) or technical errors. For our final model of timeouts, only turbine pattern, novelty and trial number were retained. Dot type (inside or outside) and whether the target was the first to be pecked were dropped from the final model. The biomimetic blade pattern was more likely to timeout than any of the other designs; the other 3 designs were not statistically significantly different from each other (Table 1; percent of trials that timed out: white = 4.19%, red = 13.42%, black = 10.45%, bio = 39.53%). Similarly, familiar blade patterns (*β* = −7.136, *Z* = −3.274, *P* = 0.001) and increasing trial number (*β* = −0.221, *Z* = −3.158, *P* = 0.0016) lowered the likelihood of a timeout. The biomimetic pattern had a significantly higher likelihood of a timeout than any other pattern and was the only pattern with a significantly higher likelihood than the white control (Fig. 3a, Table 2).

**Table 2:** Results for emmeans Tukey posthoc comparison of the wind turbine pattern's effect on the likelihood of a timeout and capture time during the blade color experiment.

Pattern A	Pattern B	Timeout				Capture time			
		Estimate	SE	*Z* value	*P* value	Estimate	SE	*t* value	*P* value
White	Red	−3.570	1.76	−2.022	0.180	−0.274	0.096	−2.862	0.0231
White	Black	−2.540	1.76	−1.443	0.473	−0.369	0.094	−3.912	0.0006
White	Bio	−11.60	3.40	−4.824	<0.0001	−0.907	0.108	−8.400	<0.0001
Red	Black	1.020	1.43	0.714	0.8917	−0.095	0.097	−0.985	0.7582
Red	Bio	−8.030	1.88	−4.270	<0.0001	−0.633	0.109	−5.819	<0.0001
Black	Bio	−9.050	2.06	−4.396	<0.0001	−0.538	0.108	−4.985	<0.0001

Negative estimates indicate that wind blade pattern A has a lower likelihood of a time out/capture time then B, while positive values indicate that A is higher than B.

As with timeouts for our final model of capture time, the wind turbine pattern shown, the novelty of the pattern, and the trial number all had a significant effect on capture time. While there were significant interactions between novelty and blade pattern, all models with interactions were a poorer fit than the 1 without interactions, as determined by likelihood ratio tests, and so the interaction term was dropped. We observed shorter delays to peck the targets for both familiar turbine patterns (ie, the pattern the bird used for training; *β* = −0.400, *t*_342.8_ = −5.03, *P* < 0.0001) and as trial number increased (*β* = −0.221, *t*_336.5_ = −6.127, *P* < 0.0001). The biomimetic pattern had a significantly higher capture time than all other blade patterns (mean times: white = 8.36 s, red = 12.05 s, black = 14.21 s and bio = 19.41 s), while the red and black treatment had a significantly higher capture time than the white but not from each other (Fig. 3b, Table 2).

Although the dot location (inside or outside of the blade-swept zone) did not statistically impact the likelihood of a bird timing out or their peck time, the outer dot tended to be chosen first, especially for the black and biomimetic blade (outer chosen first: white = 60.19%, red = 70.10%, black = 81.73% and bio = 81.08%). In the model analysing the location of the first dot choice, only the turbine pattern was retained as an effect, with both the black and biomimetic patterns significantly increasing the likelihood of the outer dot being chosen first compared with the white (Fig. 3c), but not when compared with each other or the red pattern (White–Black: *β* = −1.157, *Z* = −3.439, *P* = 0.0033; White–Bio: *β* = −1.2015, Z = −3.185, *P* = 0.0079; see Supplementary material for full table).

## Discussion

Here, we provide the first behavioral evidence that incorporating patterns onto rotating virtual objects can change the likelihood and speed with which birds approach a nearby target. This work follows a long history of evidence that contrasting patterns and colors of aposematic animals, in this case combinations of longwave colors and high achromatic contrast patterns, can increase the aversion of birds towards foreign objects (Schuler and Hesse 1985; Aronsson and Gamberale-Stille 2009; Halpin et al. 2020). Whereas other researchers have suggested that stripes can be used to improve the contrast of wind turbines (Martin and Banks 2023; Simmons and Martins 2024), we propose that they can also be used to warn birds by mimicking naturally occurring warning signals. Using patterns inspired by aposematism, we produced a biomimetic virtual wind turbine model that birds had a significantly longer time to peck targets near and the highest probability of failing to approach when compared with all other treatments. Red-striped and singular black blade patterns were also found to increase approach time, but to a lesser extent. Novelty alone is unlikely to explain our observed results for the biomimetic pattern (Barnett 1958; Marples and Kelly 1999), as the biomimetic pattern still had a higher capture time when it was familiar (ie, when the birds had previously trained on this blade appearance) compared with the white and red treatments. While birds were able to habituate to the blades, resulting in less turbine avoidance and faster peck times across sequential trials, the speed of habituation was likely heightened by the presence of a food reward associated with approaching the screen which the turbines were displayed on.

As with other computer renderings of moving objects designed to elicit flight responses in animals, such as looming objects (Carlile et al. 2006; Yilmaz and Meister 2013; Temizer et al. 2015), the birds in our experiment were observed fleeing from the novel turbines, flying back and forth across the top of their aviary as can be seen within the Supplementary Video Birdsclip. Even so, we were surprised by the extent to which our biomimetic pattern influenced the behavior of our birds, in particular the likelihood of failure to peck our target for food when exposed to our pattern. Additional experiments are required to breakdown which components of the aposematic pattern, black stripes and/or longwave pattern, resulted in this dramatic effect on the birds response to the turbines. Irrespective of pattern, the initial spike in target capture time during training when the blades started moving supports the observation that many bird species are neophobic towards wind turbines. This has been especially shown for birds with less prior exposure to turbines, such as migratory species which have been observed to modify their flight height and orientation to avoid wind farms they encounter (Schiff 1965; Johnston et al. 2014; Santos et al. 2022).

Both our biomimetic and singular black blade patterns increased the probability of birds choosing dots outside of, rather than inside of, the rotor swept area of the blades when given a choice between an inner and outer target. Black patterns have been proposed to reduce the likelihood of collision by increasing the turbine's contrast against the background, better matching the appearance of natural obstacles (eg trees) and reducing motion smearing when using offset stripes or singularly painted blades (Hodos 2003; May et al. 2020; Stokke et al. 2020; Martin and Banks 2023). The former of which is supported by the increased Michelson's contrast of the black blade and regions, see Supplementary material. Our results are unlikely to have been the result of changes in motion smear, as neither the red-striped nor the biomimetic patterns were asymmetric (would not have broken up smear). For the black blade pattern, at the spatial scale of our experiments, the turbines at 20 rpm would have had a visual angle speed of 64.0 va/s when viewed by our birds from the perch (30 cm away) and 384.0 va/s when viewed on the wire frame (<5 cm away). For a 100 m wind turbine at the same rpm, these visual angle speeds are equivalent to a viewing distance of 187.5 and 31.3 m, respectively. As motion, smear prevention strategies only influenced PERG (ie, pattern electroretinogram) between 100 and 240 va/s (Hodos 2003); at least for American kestrels (*Falco sparverius*), changes in motion smear from the black pattern would have been unlikely to influence initial responses made from the perch if great tit responses fall within a similar range.

PERG measures in response to rotating turbines have shown that black and red colors produce higher amplitude responses against blank backgrounds but not natural backgrounds, where white produces higher amplitudes (Hodos 2003). The reduced collision risk of raptors in sites where turbines have a single blade painted black (May et al. 2020) may be driven by differences in behavioral response rather than photoreceptor responses to dark moving objects compared with light. Previous experiments have shown that various vertebrate taxa are more likely to perform escape behaviors in response to dark-on-light, rather than light-on-dark, moving objects (Yilmaz and Meister 2013; Temizer et al. 2015). Differences in responses to the blades may have been driven by the elevated achromatic contrasts of our black and biomimetic treatment patterns (see Supplementary materials). If so, then it may be especially important to utilize stripes/patterns as opposed to a singular monochrome blade color due to variation in contrast under different lighting conditions, such as during the night, dawn or dusk when dark and longer (redder) wavelength colors are less contrasting (Bishop 2019). Further investigation of the influence of contrast level is needed to disentangle why birds were less likely to peck dots first within the rotor swept zone for our darker biomimetic and black bladed stimuli, as well as the influence of chromatic contrast on peck/approach time observed for our red-striped treatment.

One of the barriers to painting wind turbines and testing the effectiveness of different patterns in situ is the difficulty of re-painting wind turbines that are already operational, due to the cost of repainting, aviation regulations, manufacturer insurance and engineering concerns (May et al. 2020). The methods used here to assess the behavioral responses of birds to wind turbine patterns have the potential to be used to test a wide range of additional turbine parameters (eg, speed, pattern, number, background, etc.) far more easily and cheaply. But, further experimental validation in the field and with additional taxa is still required to ensure the changes in behavior that resulted from our biomimetic aposematic pattern are maintained at larger scales and when observed by high-risk species such as white-tailed sea eagles (*Haliaeetus albicilla*) (May et al. 2013; Balotari-Chiebáo and Byholm 2024). While large soaring birds, such as raptors, are often the target of wind turbine collision mitigation strategies, passerines, which comprise the majority of bird species, are also vulnerable to collisions with wind turbines and are often under-sampled in wind turbine studies as they are difficult to find and are quickly scavenged (Erickson et al. 2014; Nilsson et al. 2023). So even if the pattern tested only behaviorally influences passerines, the potential benefits of applying warning coloration should not be ruled out.

The primary reasons for wind turbines being painted shades of white is usually to minimize visual impact to humans, increase conspicuousness from the air, and to reduce thermal stress on the blades (Eyerly 2019). Furthermore, numerous countries have legislative constraints on the appearance of wind turbine blades that can restrict alterations to their design (May 2023). For example, in Finland, only white turbines are permitted for onshore wind farms. Visual impact on humans and aviation are also important considerations for the design and positioning of wind turbines. Despite red-striped patterns already being incorporated in some countries, their ecological effect on collision risk has yet to be formally investigated where they have been implemented. Although countries, such as South Africa, have considered using red stripes as a method for reducing raptor collisions (Simmons and Martins 2024). Yellow bands are already used for offshore wind turbines to increase conspicuousness to ships (Department of Trade and Industry [DTI], 2005), and the addition of stripes to virtual turbines has been shown to reduce the visual impact of turbines for humans when viewed from a distance (Maffei et al. 2013). Future work should also consider the potential for distant-dependent warning signals. Many aposematic patterns, such as the yellow and black markings of cinnabar caterpillars (*Tyria jacobaeae*), blur together, matching the background when viewed beyond their resolvable range, providing a distance-dependent signal effect (Barnett et al. 2018). Similar patterns could be designed to allow wind turbines to simultaneously be less conspicuous at longer distances, reducing human disruption and feasibly habituation by birds, while also providing an effective warning when observed at shorter distances.

## Conclusion

Our findings represent a crucial first step for investigating the potential of using biologically inspired warning patterns to help avoid bird collisions with wind turbines and highlight the value of using biomimicry to develop nature-inspired solutions for nature. Just as blade shapes inspired by humpback whale fins and silent owl feathers have improved turbine efficiency and reduced noise pollution, respectively (Lilley 1998; Fish et al. 2011), colors inspired by natural warning signals could provide an effective means of lowering the environmental impacts of wind turbines on bird mortality. While the biomimetic pattern we propose functions as a generic example of aposematic coloration there are many components of the pattern that have yet to be investigated such as the optimal width and number of stripes (Hodos 2003; Barnett et al. 2017, 2018), and the choice and position of the biomimetic colors (eg, does it need to be yellow/red). Other impact factors of wind turbines, such as the esthetic preferences of humans, should also be evaluated when testing future designs (Bishop 2002; Maffei et al. 2013; Sullivan et al. 2013). Likewise, countries with restrictions on wind turbine painting should consider introducing legislation to allow for wind turbines to be painted with warning patterns, even if it is only at sites close to migration flyways of vulnerable birds or in areas away from people to reduce disturbance. We hope our work encourages future exploration of the influence of features of aposematic coloration on wind turbine collision mitigation and that our experimental framework provides a means of piloting different turbine pattern regimes and testing their effect on different bird species’ behavior.

## Supplementary Material

arag039_Supplementary_Data

## Data Availability

Analyses reported in this article can be reproduced using the data provided by Hancock et al. (2026).
