# Supplementary figures and images for: Biologically inspired warning patterns deter a passerine, Parus major, from digital turbine blades
Source: Behav Ecol. 2026 Apr 20;37(4):arag039. doi: 10.1093/beheco/arag039 (PMC13178681; doi:10.1093/beheco/arag039)

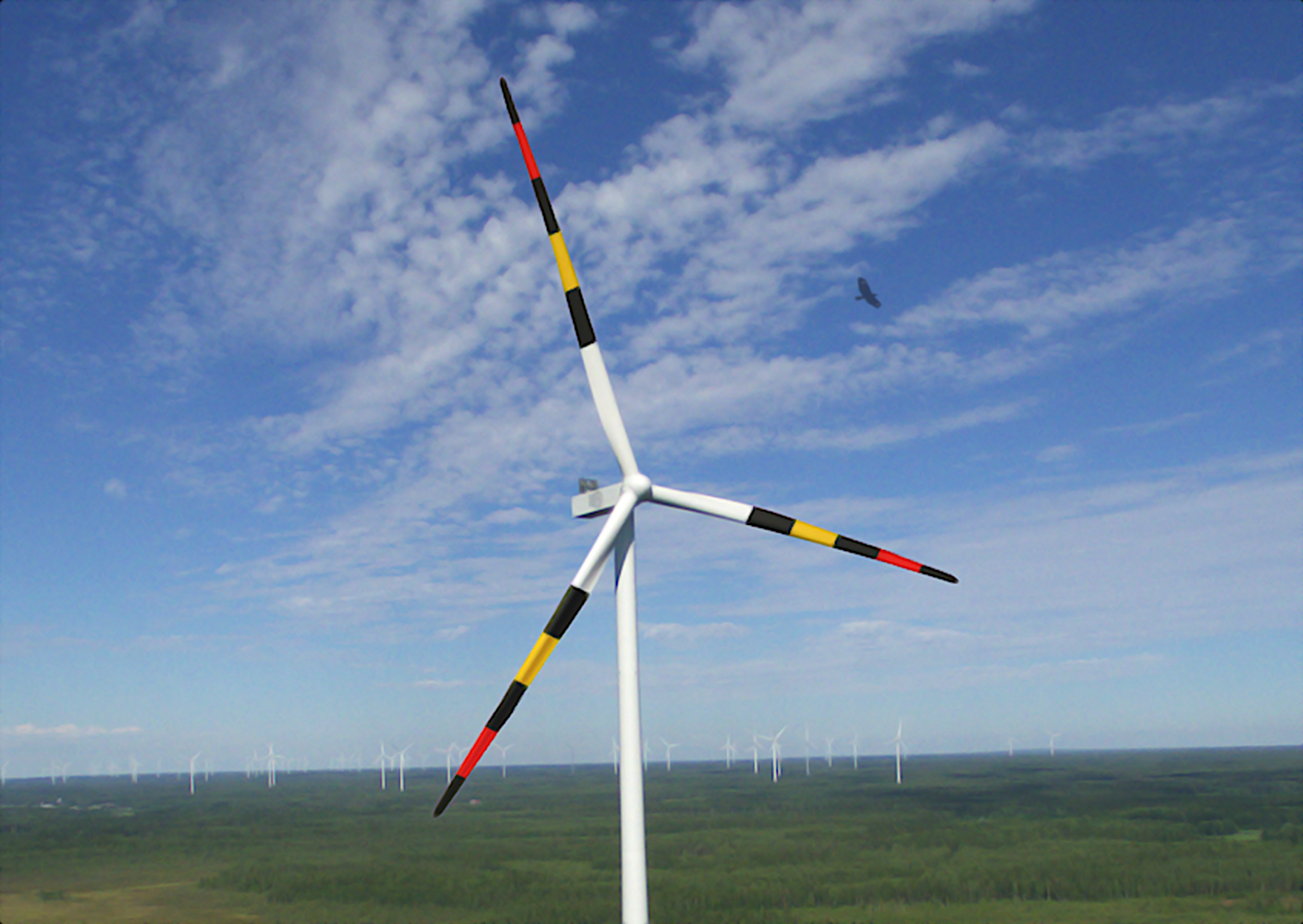

Supplement: arag039_Supplementary_Data [file arag039_supplementary_data.zip › Cover_Image_A.png]

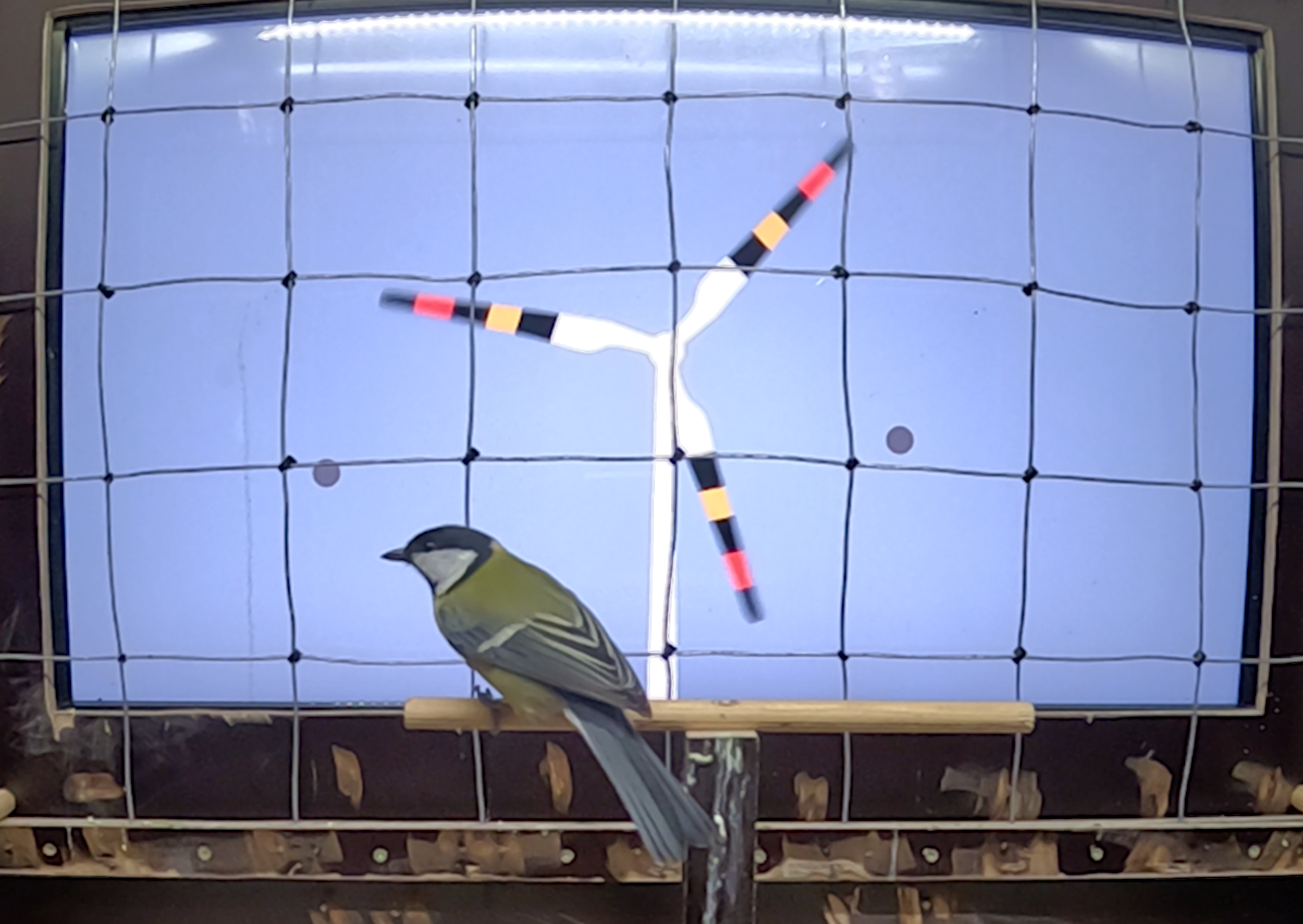

Supplement: arag039_Supplementary_Data [file arag039_supplementary_data.zip › Cover_Image_B.png]
